# Progesterone Prolongs Viability and Anti-inflammatory Functions of Explanted Preterm Ovine Amniotic Membrane

**DOI:** 10.3389/fbioe.2020.00135

**Published:** 2020-03-17

**Authors:** Angelo Canciello, Gabriella Teti, Eleonora Mazzotti, Mirella Falconi, Valentina Russo, Antonio Giordano, Barbara Barboni

**Affiliations:** ^1^Faculty of Bioscience and Technology for Food, Agriculture and Environment, University of Teramo, Teramo, Italy; ^2^Department of Biology, Sbarro Institute for Cancer Research and Molecular Medicine, Temple University, Philadelphia, PA, United States; ^3^Department for Biomedical and Neuromotor Sciences (DIBINEM), University of Bologna, Bologna, Italy; ^4^Department of Medical Biotechnology, University of Siena, Siena, Italy

**Keywords:** amniotic membrane, amniotic epithelial stem cells, progesterone, tissue culture, regenerative medicine, immunomodulation

## Abstract

Amniotic membrane (AM) is considered an important medical device with many applications in regenerative medicine. The therapeutic properties of AM are due to its resistant extracellular matrix and to the large number of bioactive molecules released by its cells. An important goal that still remains to be achieved is the identification of cultural and preservation protocols able to maintain in time the membrane morphology and the biological properties of its cells. Recently, our research group demonstrated that progesterone (P_4_) is crucial in preventing the loss of the epithelial phenotype of amniotic epithelial cells *in vitro*. Followed by this premise, it has been evaluated whether P_4_ may also affect AM properties in a short-term culture. Results confirm that P_4_ preserves AM integrity and architecture with respect to untreated AM, which showed alterations in morphology. Transmission electron microscopy (TEM) analyses demonstrate that P_4_ also maintains unaltered cell–cell junctions, nuclear status, and intracellular organelles. On the contrary, an untreated AM experienced an extensive cell death and a strong reduction of immunomodulatory properties, measured in terms of anti-inflammatory cytokine expression and secretion. Overall, these results could open to new strategies to ameliorate the protocols for cryopreservation and tissue culture, which represent preliminary stages of AM application in regenerative medicine.

## Introduction

Since the beginning of the twentieth century, researchers paid growing attention to the study of amniotic membrane (AM), even though evidences of the use of AM as a medication have been reported also in traditional Chinese medicine (Tyszkiewicz et al., 1999). Regarding scientific written reports, in 1910, the use of AM in a skin graft (Litwiniuk and Grzela, 2014) was described for the first time. Afterward, numerous studies demonstrated that the therapeutic efficacy in regenerative medicine application of the AM derives from its peculiar morphological and biological properties (Shaw et al., 2018).

Nowadays, AM, exploited as decellularized scaffold, represents a powerful medical device for the treatment of burn injuries, mechanical complications secondary to grafts, and disruption or non-healing surgical wound (Koob et al., 2015). Currently, AM is largely used in the field of regenerative medicine due to the fact that it rarely causes immunologic rejection (Jirsova and Jones, 2017). Furthermore, AM possesses great antibacterial, antimicrobial, antifibrotic, and anticancer properties (Malhotra and Jain, 2014; Jirsova and Jones, 2017; Ramuta and Kreft, 2018), even though only a marginal role of the chorion was demonstrated (Kjaergaard et al., 2001). Moreover, it has been assessed that the epithelial side of AM inhibits angiogenesis, whereas its chorionic side promotes it (Ramuta and Kreft, 2018). Indeed, the AM biological properties are mainly ascribable to the secretory abilities of its cellular components, especially of amniotic epithelial cells (AECs) that form the AM's innermost layer (Litwiniuk and Grzela, 2014) and amniotic mesenchymal stromal cells (AMSCs) that are found disperse into the extracellular matrix underneath the basement membrane (Niknejad et al., 2013). In fact, AECs are implied in the protection of embryo/fetus integrity against the maternal immune system activation due to the fact that it represents a semiallogenic body (Manuelpillai et al., 2012). This property is specifically related to the low expression of major histocompatibility complex (MHC) of class I and to the absence of MHC II and β2-microglobulin on AECs and AMSCs' surface (Miki and Strom, 2006; Barboni et al., 2012; Insausti et al., 2014). Moreover, during pregnancy or an inflammatory state, AECs secrete a plethora of anti-inflammatory cytokines, including interleukin-10 (IL-10), interleukin-4 (IL-4), and transforming growth factor-β (TGF-β) to turn off the maternal immune response (Morelli et al., 2015; Pianta et al., 2015; Barboni et al., 2018). Furthermore, AMSCs are able to block differentiation and maturation of monocytes into dendritic cells (Magatti et al., 2015). As a consequence, AMSCs induce a reduction in the production of inflammatory cytokines and an increase of anti-inflammatory cytokines (Pianta et al., 2015). Similar to AECs, AMSCs exert an antiproliferative effect on cancer cell lines by inducing cell cycle arrest in the G0/G1 phase (Caruso et al., 2012). Indeed, AECs are a rich source for cytokines that in most circumstances remain bound to the extracellular matrix even after decellularization (Sane et al., 2018). The growth factors EGF, KGF, and bFGF stimulate not only the proliferation but also the process of epithelialization of epithelial cells in the context of a wound regeneration. Moreover, TGF-β acts as extracellular matrix remodeling by stimulating fibroblasts to increase collagen production (Uchide et al., 2012). All these cytokines act in concert to orchestrate the healing process by creating the proper *milieu* for tissue regeneration.

Regenerative medicine aims to exploit the AM cellular component for its great secretory activities. Therefore, preserving AM integrity and structure becomes relevant, and any technological improvement addressed to preserve these properties is of extreme interest. Although cryopreservation is largely used for AM long-term storage, numerous evidences demonstrated that viability, anti-inflammatory, and anticancer properties could be affected by this procedure (Cooke et al., 2014; Hettiarachchi et al., 2016; Perepelkin et al., 2016; Modaresifar et al., 2017). An alternative approach is represented by the use of a fresh AM (Litwiniuk and Grzela, 2014). In this case, it would be important to identify a protocol that is able to extend the survival of AMs, thus preserving AECs' key morphological and biological features. To this regard, it was extensively demonstrated that AECs isolated from AMs lose their epithelial phenotype after few cultural passages due to TGF-β autocrine action (Alcaraz et al., 2013; Canciello et al., 2017). Recently, our research group demonstrated that progesterone (P_4_) is able to preserve AECs' epithelial native morphology and therapeutic properties, counteracting the TGF-β action (Canciello et al., 2017, 2018; Mauro et al., 2019). Indeed, P_4_ is the most representative pregnancy hormone, and it strongly affects AM physiology during the 9 months of gestation (Arck et al., 2007; Kobayashi et al., 2011; Schumacher et al., 2014). Moreover, in a mouse model, P_4_ was found to play an important role in maintaining AM tight junctions during pregnancy (Kobayashi et al., 2011). Furthermore, P_4_ is also crucial in inhibiting proinflammatory cytokine response in AMs at term or in isolated AECs (Flores-Espinosa et al., 2014; Schumacher et al., 2014; Canciello et al., 2017). However, little is known about these P_4_ effects in preterm AMs. For these reasons, the aim of the present research is to evaluate whether P_4_ is effective in preserving AM epithelial layer integrity after its isolation, by taking advantage of the sheep model (Barry and Anthony, 2008).

## Materials and Methods

### Ethic Statement

No ethic statement is required for the present research since the AMs were collected from sheep slaughtered for feed purposes.

### Amniotic Membrane Isolation

**Figure 1:**
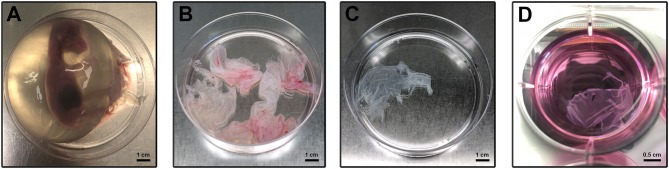
Amniotic membrane isolation and culture. **(A)** Sheep fetus isolated from the uterus according to Canciello et al. (2018) (see references). **(B)** Isolated AM pieces contain chorioallantois. **(C)** Amnion pieces appearance after mechanical removal of chorioallantois. **(D)** AM pieces during the *in vitro* culture.

The pregnant uteri were transported into the laboratory in 1 h. Immediately, the AM was collected sterile according to a validated protocol (Canciello et al., 2018; Figure 1A). Briefly, the uterus was carefully prepared, and the incision site was sterilized with denatured alcohol. In particular, for the present experiments ovine “preterm” AMs were isolated from animals at an early stage of pregnancy (1–2 months out of five of gestation) by taking into account the widespread use in regenerative medicine preclinical-related studies of this typology of cells (Barboni et al., 2018) and their widespread availability as a slaughterhouse occasional specimen. Finally, the preterm middle stage AM can be isolated with a great degree of sterility in a lab instead of the term placenta that in ovine can be exclusively collected after a natural labor since cesarean ones are rarely adopted for its high cost and convenience in sheep breeding. The uterus wall was opened with the aid of surgical forceps. Afterward, the placenta was gently separated from the uterus by manually detaching the cotyledons from caruncles and put in 1% of 10,000 UI/ml penicillin–streptomycin in sodium chloride solution (suitable for cell culture). Pieces of the AM were collected from the opposite site with respect to the umbilical cord region. Once the amnion was isolated, working under laminal flow hood, the AM was cut in macroscopic pieces in a solution of 1% of 10,000 UI/ml penicillin–streptomycin in phosphate buffered saline (PBS) without calcium and magnesium (Figure 1B). Afterward, the chorioallantois was roughly peeled off from the amnion with surgical forceps and watchmaker tweezers, working under a stereomicroscope. Working in a 10-cm petri dish, the AM was cut into smaller pieces using a 2.5 × 2.5 cm square benchmark designed on the bottom of the petri dishes (Figure 1C). Finally, the AM was cultured in non-adherent 6-well plates (Corning® Costar® Ultra-Low attachment multiwell plates, CLS3471 Sigma) (Figure 1D) in a culture medium composed of alpha minimum essential eagle medium (α-MEM, Gibco) supplemented with 20% fetal calf serum (FCS), 1% ultraglutamine (Lonza), 100 U/ml penicillin (Lonza), 100 μg/ml streptomycin (Lonza), and 2.5 μg/ml amphotericin (Euroclone) with or without 25 μM progesterone (4 pregnene-3,20-dione: P_4_, purchased from Sigma) (Canciello et al., 2017). Media were refreshed daily (Figure 1D). The AMs incubated at 38.5°C in 5% CO_2_ were analyzed at days 1, 2, and 3. In the present study, three different pieces of the AM were used for each experimental condition (experimental triplicates). Moreover, the experiments were carried out using AM specimens derived from at least three different animals (biological triplicates). Early gestational sheep uteri (1–2 months of gestation) were collected from Adult Appeninca bred animal (age from 2 to 4 years old) approximately 50 kg in weight grown in extensive farming. Unlike the human AM, which can be collected only at labor, the ovine AM can be easily obtained from different stages of gestation (from early to late at labor) offering the possibility to have the availability of samples with different biological characteristic. Finally, it is well-known that the ovine placenta can be considered as a reliable translational mammal model for human pregnancy (Barry and Anthony, 2008).

### Viability Analysis

In parallel, AM pieces were used for assessing cell viability at each time point. To this aim, the vital quantifiable fluorescent Calcein AM (C1359 Sigma-Aldrich), propidium iodide (P4864 Sigma-Aldrich), and 4′,6-diamidino-2-phenylindole (DAPI) staining (Vectastain) were used. Briefly, working in the dark, Calcein AM, DAPI, and propidium iodide were consecutively added to the culture medium (45, 5, and 2 min, respectively, before performing the subsequent analysis) according to the manufacturer's instructions. For the fluorescence analysis, Nikon A1r confocal microscope interfaced to a computer workstation, provided with NIS-Elements 4.4 software (for images acquisition) and with NIS-Elements Advanced Research imaging software (for post-processing analysis), was used. Quantification of Calcein AM, propidium iodide, and DAPI fluorescence was performed by using the image-processing software ImageJ, version 1.40g (http://rsbweb.nih.gov/ij). The viability of the fresh AM was taken as 100%. Each experimental group was analyzed in triplicate, and the relative levels of fluorescence were obtained from 10 random fields.

### Histological Investigation

AM pieces were further divided into three to five portions and fixed in 4% paraformaldehyde for 48 h, washed in dH_2_O, dehydrated, and embedded with consecutive passages in dehyol 95%, dehyol 100%, xylene, and hot paraffin, each step lasting 1 h.

Serial paraffin sections of 5-μm thickness were collected on poly-L-lysine-coated slides and sequentially processed for investigations. In more details, AMs were serially sectioned and subjected to hematoxylin (Merck, Darmstadt, Germany)/eosin (Merck) staining in order to identify the microarchitecture of the epithelial layer and to verify morphologically the degree of tissue integrity. Morphological analyses were performed with an Axioscop 2plus epifluorescence microscope (Zeiss, Oberkochen, Germany) equipped with a cooled color charge-coupled device camera (CCD; Axiovision Cam, Zeiss) interfaced to a computer workstation and provided with an interactive and automatic image analyzer (Axiovision, Zeiss).

### Transmission Electron Microscopy

At each experimental time and condition, AM samples were fixed with 2.5% glutaraldehyde in 0.1 M PB for 2 h at 4°C and were subsequently washed post-fixed with 1% OsO_4_ in 0.1 M PB for 1 h at room temperature (RT). After several washes in PBS, the samples were dehydrated in an ascending alcohol series (25, 50, 70, 90, 100%) and embedded in Epoxy with resin (Fluka, Sigma Aldrich, St. Luis, Missouri, USA). Afterward, the fixed AMs were sectioned in a Reichert Jung FC 4/E (Leica, Wien, Austria) ultramicrotome. Sections of 90 nm were mounted on grids and stained with uranyl acetate and lead citrate for 10 min at RT. The analysis was carried out by CM10 Philips TEM (FEI Company, Eindhoven, The Netherlands) at an accelerating voltage of 80 kV. The images were recorded using a Megaview III digital camera (FEI Company, Eindhoven, The Netherlands).

### Amniotic Membrane Cytokines Basal and Lipopolysaccharide-Induced mRNA Expression

In order to verify the ability to preserve AM functionality during the culture, the expression levels of cytokines were analyzed at time points 1 and 3 (days) with or without exposing the tissue to an inflammatory stimulus mimicked by lipopolysaccharide (LPS, from *Escherichia coli* 055:B5, purchased from Sigma) (1 μg/ml for 24 h). More in detail, mRNA expression levels of *IL10, IL4*, and *TGF*β mRNA expression were compared between CTR and P_4_-treated AM. To this aim, total mRNAs were extracted by using TRIzol (Sigma) according to the manufacturer's instructions. Integrity and size distribution were evaluated by 1% agarose gel electrophoresis and GelRed staining (Biotium). Quantification of total mRNA samples was assessed by using Thermo Scientific NanoDrop 2000c UV-Vis spectrophotometer at 260 nm. Digestion of genomic DNA was carried out by DNaseI (Sigma) exposing the samples for 15 min at RT. cDNA was synthetized from 1 μg of total RNA of each sample, which was used for reverse transcription reaction with Random Hexamers primer and Tetro Reverse Transcriptase (Bioline) at a final volume of 20 μl according to the manufacturer's instructions. Afterward, Real-time qPCR analysis was performed by using SensiFAST™ SYBR Lo-ROX kit (Bioline) by adjusting the manufacturer's instruction to a final volume of 15 μl, by using the following primers: IL-10 forward 5′-CCAGGATGGTGACTCGACTAG-3′ and reverse 5′-TGGCTCTGCTCTCCCAGAAC-3′; IL-4 forward 5′-AAGCCCTCAGCTAAGCATGT-3′ and reverse 5′-AGGCATCACAGGCTCAAGTC-3′; TGF-ß forward 5′-GAAGTCTAGCTCGCACAGCA-3′ and reverse 5′-CACGTGCTGCTCCACTTTTA-3′; GAPDH forward 5′-TCGGAGTGAACGGATTTGGC-3′ and reverse 5′-CCGTTCTCTGCCTTGACTGT-3′; 40S Ribosomal protein S8 (RBS8) forward 5′-GCTTCTTGCATGCATCGCTT-3′ and reverse 5′-CCTTTCCGGGCCTTGATCTT-3′. The reaction was carried out with 7500 Fast Real-time PCR System (Life Technologies) by using the two-step cycling protocol for 40 cycles (10 s at 95°C for denaturation and 30 s at 60°C for annealing/extension) followed by melt-profile analysis (7500 Software v2.3). The relative expression level of mRNA was calculated by the ΔΔCt method.

### Amniotic Membrane Cytokine Release in the Culture Medium

The integrity of the AM during the culture in the presence or absence of P_4_ was assessed by analyzing the release of anti-inflammatory cytokines into the cultural medium. To this aim, Nori Sheep ELISA Kits (Genorise Scientific, Inc.) were used to quantify IL-10, IL-4, and TGF-β. Standard and culture media samples obtained by CTR and P_4_-treated AM were added to the microtiter plate wells with horseradish peroxidase (HRP) conjugated antibodies and processed according to the manufacturer's instructions. The optical density of each well was determined by using a microplate reader set to 450 nm and subtracting the corresponding reading at 540 nm for each well.

### Statistical Analysis

Data reported in this paper were obtained from three early-gestation fetuses. The results are the mean (±SEM) of at least three independent experiments, each performed in triplicate. All statistics were performed using Prism 6 (GraphPad). Two-way ANOVAs with multiple *t*-test comparisons were performed on data sets with two independent variables. At least a *p* < 0.05 was considered statistically significant.

## Results

### Progesterone Preserves the Architecture of Amniotic Membrane During 3 Days of Culture

In order to evaluate the effects of P_4_ in maintaining AM architecture during *in vitro* culture (Figure 1), CTR and P_4_-treated AMs were collected after 1, 2, and 3 days and subjected to hematoxylin–eosin staining. In order to determine the concentration of P_4_ to use in the experiments, serial dilutions of P_4_ (0.001, 0.01, 0.1, 1, 2.5, 5, 10, 25, 50, and 100 μM) were tested (data not showed). As a result, only 25 μM of P_4_ showed significant effects on the preservation of AM cell viability and tissue architecture. For this reason, 25 μM of P_4_ was the concentration adopted in the following experiments. Besides, this concentration of P_4_ was in accordance with data presented in literature (Kobayashi et al., 2011; Canciello et al., 2017).

**Figure 2:**
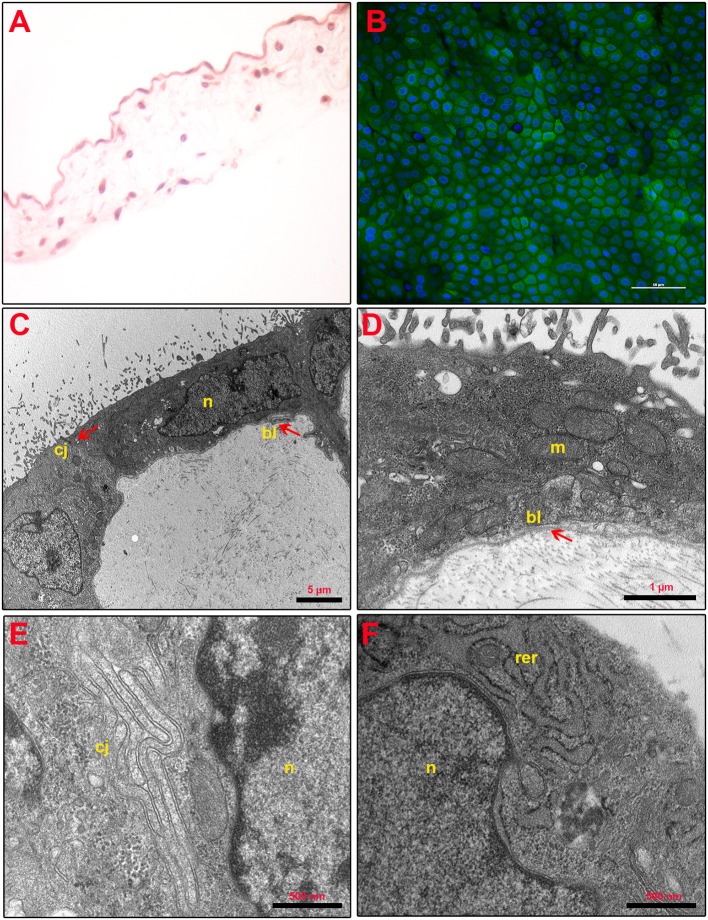
Fresh AM histology, viability, and ultrastructure. **(A)** Fresh AM pieces were subjected to hematoxylin–eosin staining to evaluate their membrane architecture. Magnification 40×. **(B)** Fresh AMs were subjected to staining by using Calcein AM (green), DAPI (blue), and propidium iodide (red) to assess cell viability and mortality. Magnification 40×. **(C–F)** Ultrastructure of fresh AM shows cell morphology **(C)**, nuclear details (n) **(C,F)**, apical specializations and basal lamina (bl) **(D)**, cell–cell junctions (cj) **(E)**, and mitochondria (m) and RER (rer) **(F)**.

**Figure 3:**
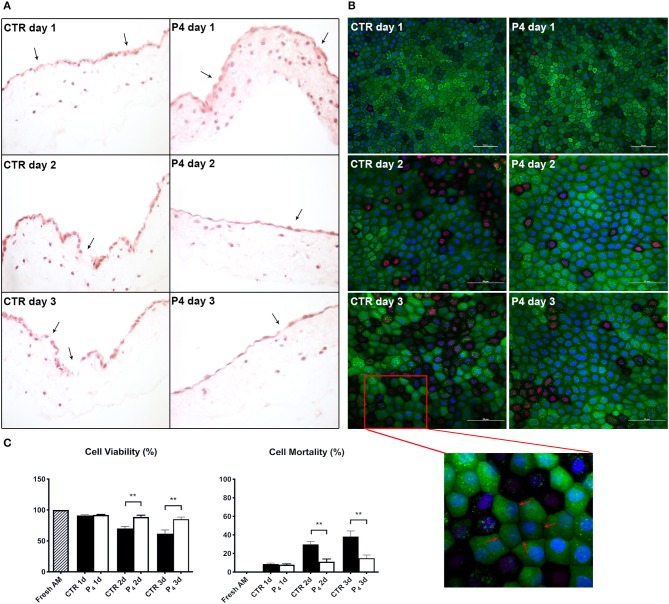
AM histology, morphology, and viability. **(A)** AM pieces were cultured up to 3 days in the presence (P4) or in the absence (CTR) of P_4_ supplementation and then were subjected to hematoxylin–eosin staining to evaluate their membrane architecture. **(B)** CTR and P4-treated AM were *in vitro* cultured up to 3 days and then were subjected to staining by using Calcein AM (green), DAPI (blue), and propidium iodide (red) to assess cell viability and mortality. Representative 40× magnification is shown for CTR and P4 day 1 acquisition; 60× magnification or days 2 and 3. Scale bar: 50 μm. **(C)** Quantification of Calcein AM, propidium iodide, and DAPI fluorescence was performed by using image-processing software ImageJ, version 1.40g (http://rsbweb.nih.gov/ij). The viability of freshly isolated AM was taken as 100%. Each experimental group was analyzed in triplicate, and the relative levels of fluorescence were normalized by the mean level of fluorescence obtained from three replicates of the medium alone.

As shown in Figure 2A, the structure of fresh *amnion* is composed of mucosa recognizing epithelial cells organized in a monolayer and a submucosa where mesenchymal cells are widespread among an abundant avascular extracellular matrix. The cultural condition dramatically affected the AM morphology. Indeed, AM incubated adopting standardized medium (CTR) precociously displayed signs of tissue disorganization (day 1) with gaps among the cells that interrupted the continuity of the epithelial sheet (Figure 3A). AM architecture was further compromised when at days 2 and 3 it became frequent to detect detachments of the epithelial layer from the underlying basal membrane (Figure 3A). Conversely, the P_4_-treated AM compared with the fresh AM (Figure 2A) showed an overall higher integrity with a preserved microarchitecture persisting during the culture (Figure 3A). In particular, no structural alterations were observed in the P_4_ AM until day 3, when only rare membrane breaks occurred on the epithelial layer (Figure 3A).

### Progesterone Increases Amniotic Membrane Cell Viability Over 3 Days of *in vitro* Culture

Furthermore, the positive P_4_ influence on AM culture was further confirmed by analyzing cell viability. Calcein AM, propidium iodide, and DAPI staining were performed on the fresh AM, P_4_-treated AM, and CTR AM in order to investigate cell viability and cell mortality and to quantify the total number of cells (nuclei), respectively (Figures 2B, 3B). Calcein staining confirmed that the CTR AM at day 1 displayed a high degree of membrane impairing with respect to the fresh AM (Figure 2B) with several epithelial cells having lost the cobblestone-like structure and cell–cell contact (Figure 3B). Moreover, the incidence of cell death (red nuclei) is significantly higher in the CTR AM at days 2 and 3 with respect to the P_4_-treated AM. In addition, the percentage of dead cells increased approximately by five times passing from day 1 to day 3 of culture (from 8.8 ± 1.0 to 38.3 ± 6.0%, respectively; Figure 3C). By contrast, the P_4_-treated AM during the whole cultural interval maintained a good level of membrane integrity characterized by a preserved epithelial cell–cell contact and a steady cell viability (from 92.0 ± 1.5 to 85.1 ± 3.5% at day 1 and day 3, respectively; Figure 3C).

### Progesterone Improves Amniotic Membrane Ultrastructure During the Incubation

**Figure 4:**
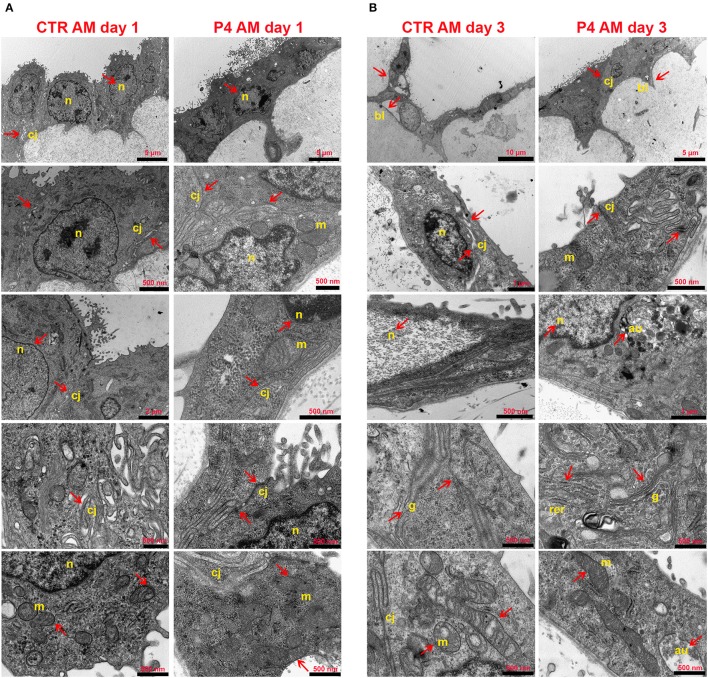
AM ultrastructural analysis. Ultrastructure of AM pieces after 1 **(A)** and 3 **(B)** days of culture in the presence (P4) or in the absence (CTR) of P_4_ supplementation was evaluated by using TEM. **(A)** From the top to the bottom of the panel, cell morphology, nuclear details (n), apical and basal specializations, cell–cell junctions (cj), and mitochondria (m) are shown. **(B)** From the top to the bottom of the panel, cell morphology, cell–cell junction (cj), nuclear details (n) and basal lamina (bl), RER (rer), Golgi (g), and mitochondria (m) are shown.

In order to investigate the effects of P_4_ treatment on AM ultrastructure, pieces of the AM cultured in the absence (CTR AM) or in the presence of P_4_ were analyzed at days 1 and 3 (Figure 4). Ultrastructural evaluation was conducted also on the fresh AM (Figures 2C–F). TEM analysis showed that the CTR AM already at day 1 started to show signs of disorganization with enlarged cells displaying a surface covered by cytoplasmic protrusions and a euchromatic nucleus. At higher magnification, the Golgi apparatus was frequently disassembled and surrounded by several small vesicles. Rough endoplasmic reticulum (RER) cisternae were numerous and enlarged, showing inside a moderately electron dense material. A high number of mitochondria with evident internal cristae were also observed. Moreover, a great number of cell junctions and desmosomes were observed (Figure 4A).

Conversely, the P_4_-treated AM still showed at day 1 a well-conserved morphology with the epithelial layer composed of oblong-shaped cells with some apical specializations (Figure 4A) similar to those found in the fresh AM (Figure 2C). A higher number of digitiform intercellular junctions were detected between epithelial cells (Figure 4A). Interestingly, in correspondence with cell–cell junctions, the P_4_-treated AM showed similar interdigitations to those found in the fresh AM (Figure 2E). Furthermore, the lateral cell membrane showed complex interdigitations in association with some desmosomes. A well-developed basal labyrinth was noticeable in multiple areas of the membrane. Nuclei were characterized by various shapes, and some electron dense areas with an envelope were easily detectable. Similar to the fresh AM (Figures 2D,F), the cytoplasm of the P_4_-treated AM displayed a RER with cisternae showing moderately electron dense material and several mitochondria with evident cristae (Figure 4A). At middle magnification, a widely developed Golgi apparatus surrounded by several secretory vesicles was observed (Figure 4A).

The epithelial layer of the CTR AM at 3 days showed a high degree of disorganization. The cells showed an abnormal shape and nuclei with high euchromatic and several vesicles. Several autophagolysosomes characterized their cytoplasm. Distinct signs of apoptosis were detectable in almost every cell. The mitochondria were dramatically decreased, the cristae were clearly dilatated, and some mitophagy vesicles were detected. The RER cisternae were highly dilatated and the Golgi apparatus was not detectable. A reduced number of intercellular junctions were detected with a considerable increase in the average intercellular spaces (Figure 4B).

On the other hand, the morphology of the P_4_-treated AM was still preserved with elongated covering epithelial cells displaying easily detectable nuclei with a variable number of nucleoli. The basal labyrinth was still found in almost every section. Rare autophagy vesicles were observed. The mitochondria numbers were almost unchanged even if they appeared smaller and with enlarged cristae as well as the RER cisternae. Furthermore, for the first time, some cells presented cytoplasmic protrusions on the apical edge (Figure 4B).

### Progesterone Changes the Basal and Lipopolysaccharide-Induced Amniotic Membrane-Derived Anti-inflammatory Cytokines mRNA Expression

**Figure 5:**
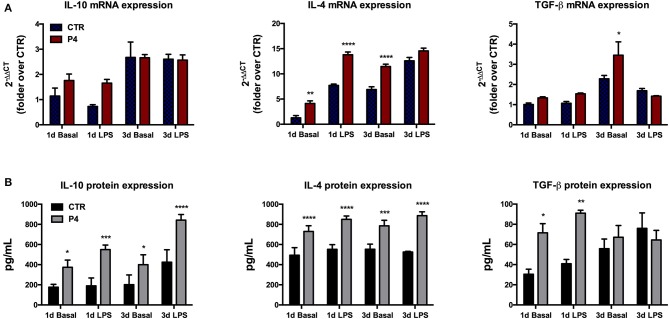
Anti-inflammatory cytokine mRNA expression and culture medium content. **(A)** Comparison between basal (blue bars) and LPS-induced (red bars) IL-10, IL-4, and TGF-β mRNA expression in CTR and P4-treated AM, after 1 (1 d) and 3 (3 d) days of *in vitro* culture. **(B)** Comparison between basal (black bars) and LPS-induced (gray bars) IL-10, IL-4, and TGF-β content in CM collected from CTR and P4-treated AM, after 1 (1 d) and 3 (3 d) days of *in vitro* culture. Data are the mean ± SEM, from *n* = 4 independent experiments performed in triplicate. *p < 0.05, **p < 0.01, ***p < 0.001, and ****p < 0.0001.

The regenerative properties of the AM have been related to their paracrine influences exerted in host damaged tissues aimed to release several anti-inflammatory and immunomodulatory cytokines. Therefore, in order to verify the effects of P_4_ on these AM abilities, the expression levels of anti-inflammatory cytokines (*IL-10, IL-4*, and *TGF-*β) were compared between the CTR and P_4_-treated AM. In detail, the basal expression levels of anti-inflammatory cytokines were analyzed in comparison to those obtained after the exposure to bacterial LPS (to simulate an inflammatory-like condition) (Figure 5A). As shown in Figure 5A, at day 1 the basal cytokine expression was similar in the CTR and P_4_-treated AM except for *IL-4* mRNA that was up-regulated in the presence of P_4_ (3.2-fold increase, *p* < 0.01). The higher immunomodulatory activity of the P_4_-treated AM became evident at day 3 when an overexpression of both *IL-4* and *TGF-*β (*p* < 0.0001 and *p* < 0.05 vs. CTR, respectively) was recorded (Figure 5A).

On the contrary, no differences were recorded upon LPS stimulation in cytokine expression between the CTR and P_4_-treated AM with the only exception for *IL-4* at day 1 that showed a significant up-regulation (*p* < 0.0001 vs. CTR) in the P_4_-treated AM (Figure 5A).

### P_4_-Treated Amniotic Membranes Increase the Anti-inflammatory Cytokine Release in Culture Medium

P_4_ treatment strongly improved the AM ability to release anti-inflammatory cytokines in the culture medium (CM). In more details, the basal levels IL-10, IL-4, and TGF-β recorded in the CM of the AM cultured for 1 day in the presence of P_4_ were significantly higher than those recorded from the CTR AM (2.1-, 1.5-, and 2.3-fold increase with *p* < 0.05, *p* < 0.001, and *p* < 0.05, respectively; Figure 5B). The analysis of the CM cytokine content at day 3 showed that the P_4_-treated AM maintained a higher basal secretion of IL-10 (2.0-fold, *p* < 0.05) and IL-4 (1.4, *p* < 0.001), while the levels of TGF-β were similar to those of the CM collected from the CTR AM (Figure 5B).

Analogously, the P_4_-treated AM released a significantly higher concentration of IL-10 (2.9-fold, *p* < 0.001), IL-4 (1.5-fold, *p* < 0.0001), and TGF-β (2.2-fold, *p* < 0.01) upon LPS stimulation at day 1 (Figure 5B). The greater anti-inflammatory activity of the AM cultured with P_4_ was also confirmed at day 3 when significantly high levels of IL-10 and IL-4 were recorded in their CM collected after exposure to LPS. Conversely, the basal levels of anti-inflammatory cytokines recorded at day 3 in the CM of the CTR AM were similar to those analyzed at day 1, except for the TGF-β content, which showed a slight increase (Figure 5B).

## Discussion

The present study proposes a novel cultural approach able to maintain *in vitro* the morphological and biological properties of the AM. The results confirmed that the supplementation of P_4_–the main pregnancy hormone—improved the morphological features of the cultured AM. Interestingly, P_4_ not only influences the integrity of AM microarchitecture but is also able to enhance its immunomodulatory/anti-inflammatory activity, thus preserving a key biological function that complies with the regenerative properties of this fetal tissue. Indeed, it is well-known that the AM is able to exert a tunable paracrine function aimed to physiologically protect the growing fetus. This protective function is exerted through the steady basal secretion of anti-inflammatory cytokines that can be further modulated in response to inflammatory stimuli, such as LPS (Flores-Espinosa et al., 2014). Indeed, these immunomodulatory properties make the AM of great interest in the field of regenerative medicine due to the fact that they can contribute to ameliorate the wound healing process and to reduce the tissue inflammatory state. For these reasons, AMs find a number of applications in regenerative medicine. In fact, beside its large use in ophthalmic surgery, AM transplantation has been successfully used for skin burns, diabetic foot ulcer, and tendon and nerve repair and also for several sport medicine applications (Liu et al., 2010; Riboh et al., 2016; Zhang et al., 2019).

Here, it has been demonstrated that the AM responsiveness to LPS was progressively lost during the culture when the incubation was performed adopting standardized conditions. Only the supplementation of P_4_ was able to revert this undesirable event by enhancing both basal and LPS-induced anti-inflammatory cytokine AM secretory function.

The paracrine function of the AM derives mainly from the biological properties of amniotic epithelial cells (AECs), an emerging and promising source of placental stem cells (Miki and Strom, 2006). Similar to other stem cells, AECs can be quite heterogenous in terms of phenotype and biological properties, which are strongly influenced by several factors: their source (Miki and Strom, 2006; Mattioli et al., 2012; Murphy and Atala, 2013; Koike et al., 2014); the *in vitro* manipulation procedures (Barboni et al., 2012; Canciello et al., 2017, 2018; Vitucci et al., 2018); the site of injection (Caruso et al., 2012); and the dialogue occurring after transplantation between cells and host tissues (Manuelpillai et al., 2012; Mauro et al., 2016). A reduction of native epithelial phenotype may be the consequence of inadequate procedures occurring after stem cell isolation (Stadler et al., 2008; Caruso et al., 2012; Murphy and Atala, 2013), such as the cultural (e.g., growth factors, hormones) and environmental conditions (e.g., pH, O_2_ levels) that both can drastically reduce the biological properties of the cells even after short-term *in vitro* culture (Mendelson and Condon, 2005; Bilic et al., 2008; Pratama et al., 2011; VandeVoort et al., 2011; Giarnieri et al., 2015; Jeon et al., 2016). Therefore, researchers have paid great attention to standardize cultural protocols (Pratama et al., 2011; Caruso et al., 2012; Manuelpillai et al., 2012; Barboni et al., 2014; Canciello et al., 2018), first to facilitating stem cell use among scientific groups and secondarily to preserve or even improve their native biological regenerative potential (Moodley et al., 2010; Manuelpillai et al., 2012; Barboni et al., 2013; Canciello et al., 2017).

Recently, our research group demonstrated that P_4_ supplementation to AECs *in vitro* culture is essential to prevent the epithelial to mesenchymal transition (Canciello et al., 2017) that occurred spontaneously during the *in vitro* cell amplification. This event leads in culture to the precocious loss of the epithelial phenotype and to the acquisition of mesenchymal one. Along with the maintaining of AECs' epithelial phenotype, P_4_ treatment also preserved some crucial AECs' properties such as their stemness and immunomodulatory properties (Canciello et al., 2017). A similar protective effect of P_4_ was confirmed in the present research on the AM.

The P_4_ in culture seems to maintain its physiological role that during pregnancy is addressed to the fetus, uterus, and fetal *annexa* (Silver, 1994; Kobayashi et al., 2011; Abdulkareem et al., 2012). Numerous evidences demonstrated an involvement of P_4_ in controlling *amnion* homeostasis through the maintaining of tight junctions—essential for the proper epithelial monolayer assembling—(Kobayashi et al., 2011), the modulation of its receptivity (Mendelson and Condon, 2005; Flores-Espinosa et al., 2014), and the regulation of inflammatory cytokine secretion (Loudon et al., 2003).

In more details, P_4_ seems to contribute to the prenatal formation of tight junctions in the AM, a fundamental morphological feature (Kobayashi et al., 2011). This occurs especially during mid-pregnancy stage, when the AM and AECs exhibit high expression of progesterone receptor (PR) through which P_4_ induces the upregulation of claudin-4 and occludin, two main components of the tight junctions (Kobayashi et al., 2011). Interestingly, P_4_ preserves unaltered the integrity of the cell–cell junction that both in fresh and in P_4_-treated AMs are located near profound interdigitations of the lateral membrane. This seems to be a characteristic of the amniotic epithelium, which is dramatically lost when AMs are cultured *in vitro* without P_4_ supplementation.

In accordance with these evidences, here it has been demonstrated that P_4_ supplementation to AM *in vitro* culture can be exploited to prolong AM integrity and cell viability up to 3 days of culture. On the contrary, the AM cultured in the absence of P_4_ rapidly lost the integrity of the epithelial structure with the dramatic drop of cell viability. Moreover, the P_4_-treated AM epithelial layer presents common morphological features with the fresh AM, even after 3 days of culture. In detail, both fresh and P_4_-treated AMs are composed of oblong and flattened epithelial cells with well-visible nuclei. Conversely, it seems that the absence of P_4_ in the culture affects the morphology of AM epithelial cells, which appear more rounded and with irregular apical surfaces. Besides, the microarchitecture of the epithelial layer was rapidly compromised with the appearance of gaps or interruptions in the epithelial sheet and severe cell detachments from the underlying basal membrane. The protective effects of P_4_ on the AM were, on the contrary, demonstrated by the preservation of the epithelial monolayer microstructure over the 3 days of culture with only rare membrane breaks in the epithelial sheet. In accordance with these findings, Kumar et al. (2015) recently have demonstrated that P_4_ plays a protective role during AM *in vitro* culture inhibiting the production of GM-CSF, which, in turn, induces fetal membrane weakening at labor (Kumar et al., 2015). On the basis of these evidences, P_4_ plays a crucial role in the homeostasis of the AM, and its administration to the culture medium could recreate *in vitro* a more physiological environment able to reproduce the signaling that AM cells experienced during the 9 months of gestation. This aspect, in part, could explain the high level of cell death found in the AM cultured without P_4_. However, further experiments are needed in order to understand the role of P_4_ in preserving cell viability in a long-term AM culture.

The positive influence of P_4_ on the persistence of intercellular junctions is reinforced by the TEM data that confirmed an increased degree of cell interconnections and a more complex organization of the epithelial layer. Only under P_4_ cultural condition AMs were able to conserve the basal labyrinth and the interdigital junction by reducing the desmosome-like structures that may be essential to control the import and export of bioactive molecules through this avascular membrane. Interestingly, tight junctions seem to be enriched near the apical surfaces of the epithelial cells, whereas on the lateral membrane, profound interdigitations are prevalent. Conversely, untreated AMs show a reduction of these interdigitations, which appear looser. It is possible that the increase in desmosomes described after the first day of *in vitro* culture could be due to the loss of lateral contact between adjacent cells. Indeed, the morphological asset of the untreated AM seems to reinforce the idea that a drastic reduction of the cellular interdigitations in favor of an increasing number of desmosomes may represent an early sign of *amnion* dysfunction. A similar hypothesis was proposed by Wintour et al. (1978) who identified that an increase in P_4_ during sheep gestation modifies the junctional complex of the epithelium cells (Wintour et al., 1978).

P_4_ is capable, at the same time, to improve AM cell viability confirmed either by immunofluorescence or ultrastructural observations. In addition, epithelial cells belonging to the AM cultured in the presence of P_4_ preserved unaltered their shape and cytology. Their cytoplasm appeared electron dense, and only few autophagy vesicles were present up to 3 days of culture. Instead, the AM cultured in the absence of P_4_ showed precocious signs of cellular death, such as the presence of autophagosomes, cell membrane ruptures, and heterochromatin-rich nuclei. Intriguingly, P_4_ seems also to play a role in regulating AM cell metabolism. Indeed, the persistence of a well-developed SER and Golgi apparatus was a steady record of the epithelial cells of the AM incubated with P_4_, suggesting a positive correlation with an increased lipid synthesis.

It has been demonstrated that the AM increases its immunomodulatory and anti-inflammatory properties upon an inflammatory stimulus, which is likely to prevent the risk of a premature abortion (Flores-Espinosa et al., 2014). Likewise, AECs and AMSCs also show an increase in their native immunomodulatory properties by secreting high levels of anti-inflammatory cytokines when stimulated with bacterial LPS (Canciello et al., 2017; Kong et al., 2018). In the present study, it was not possible to distinguish between the contribution of the two AM cell types (AECs and AMSCs) even if, considering the prevalence of AECs in the AM structure, it would be wise to think that the increase in anti-inflammatory cytokines' mRNA expression and releases recorded after LPS stimulation may be mainly attributable to the epithelial layer. Nevertheless, in the present study, the ILs' profile was aimed to test and compare the functionality of different cultured conditions on the AM (CTR and P_4_-treated AM).

Here, indeed, it has been demonstrated that P_4_ improves both the mRNA expression and the secretion of IL4, IL10, and TGF-β in the AM. However, it remains to be demonstrated if this positive result was determined by a direct action of P_4_ on the intracellular pathways mediating cytokine synthesis or if it was an indirect consequence of the higher cell viability induced by progesterone supplementation. For instance, it is well known that P_4_ played a role in promoting maternal–fetal tolerance by affecting the cytokine production of the maternal immune placental cells (Arck et al., 2007). However, independently of the mechanism of action, this finding reinforces our previous results indicating a positive role of P_4_ in the protocols of AECs *in vitro* expansion. In particular, P_4_ treatment induced a great increase in *IL4, IL10*, and *TGF-*β mRNA levels and protein secretion with a concomitant inhibition of inflammatory cytokines (*IL-1*β, *IL6*, and *IL12*) in AECs (Canciello et al., 2017). In the present study, the anti-inflammatory cytokine profile of the AM was analyzed to report the influence of the two cultural conditions (with or without P_4_) in preserving tissue functionality. Indeed, AM responsiveness to an inflammatory stimulus, such as LPS, could give some insights to understand the functional status of the tissue. In this regard, an increase of anti-inflammatory cytokine mRNA expression indicates that the P_4_-treated AM—along with the cells that compose this tissue—preserved tissue functionality with respect to the untreated AM, which are less sensitive to LPS stimulation. On the other hand, it could not be excluded that the reduction of anti-inflammatory cytokine secretion recorded in the CTR AM could be related to massive cell death and thus to the presence of less functional cells that are able to respond to LPS stimulation. However, P_4_, by preventing this cell death, is in turn able to maintain AM responsiveness to LPS and to induce an increase in anti-inflammatory cytokine expression. Therefore, the variations observed in AM cytokine secretion after P_4_ treatment need further experiments to be confirmed. Finally, although pro-inflammatory cytokine expression was not evaluated in this study, the inverse role of P_4_ in modulating the anti- and pro-inflammatory cytokines was extensively studied by (Flores-Espinosa et al., 2014).

The practical advantage to preserve in time the microarchitecture and functionality of the AM leads to an improvement in AM use for regenerative medicine practices and also to an increase in the chances to isolate AECs with well-preserved biological properties. To this regard, despite these latter abilities, the AM is also considered for its structural properties. Indeed, after de-epithelialization and sterilization, the AM is used as a scaffold for the peculiar properties of its extracellular matrix (Litwiniuk and Grzela, 2014). For this reason, from the second half of the twentieth century, the AM became one of the first biomaterials used in tissue engineering as a scaffold for cell growth, thus increasing the applications in ophthalmology (ocular surface reconstruction), thoracic surgery (pleural and pericardial closure), and in the treatment of severe burns and chronic ulcers (Meller et al., 2011; Manuelpillai et al., 2012; Keerthi et al., 2015). On the other hand, whole pieces of the non-decellularized AM are used to treat several pathologic conditions, such as large skin losses, shallow wounds of the skin surface, mechanical injuries, or toxic epidermal necrolysis (Klama-Baryla et al., 2018). In recent years, researchers are studying new possible clinical approaches for the use of the AM in regenerative medicine. Indeed, small pieces of the AM have shown promising results in the treatments of cardiac ischemia and liver fibrosis by ameliorating both the tissue damage and the physiological functions of the compromised organs (Manuelpillai et al., 2012).

The results presented in this study provide evidence for the protective role played by P_4_ in maintaining and preserving *in vitro* the morphological and biological features of the AM. Furthermore, given the wide use of the AM in regenerative medicine, these findings could help to improve AM harvesting, *in vitro* culture methods, long-term preservation protocols (cryopreservation, lyophilization), and eventually the AM performances in clinical applications.

## Data Availability Statement

The datasets generated for this study are available on request to the corresponding author.

## Ethics Statement

Ethical review and approval was not required for the animal study because none ethic statement is required for the present research since the amniotic membranes were collected from sheep slaughtered for feed purposes.

## Author Contributions

AC conceived the project, took the lead in experiments, data evaluation, and manuscript writing. BB supported the manuscript writing, supervised data analysis, and co-financed the research. VR supported placenta sample selection and the histological and viability analyses. GT and EM prepared the samples for TEM analysis. AG and MF supervised the data analysis and manuscript writing.

### Conflict of Interest

The authors declare that the research was conducted in the absence of any commercial or financial relationships that could be construed as a potential conflict of interest.
